# Clinical and Recent Patents Applications of PD-1/PD-L1 Targeting Immunotherapy in Cancer Treatment—Current Progress, Strategy, and Future Perspective

**DOI:** 10.3389/fimmu.2020.01508

**Published:** 2020-07-07

**Authors:** Libin Guo, Ran Wei, Yao Lin, Hang Fai Kwok

**Affiliations:** ^1^Cancer Centre, Faculty of Health Sciences, University of Macau, Avenida de Universidade, Taipa, China; ^2^Key Laboratory of Optoelectronic Science and Technology for Medicine of Ministry of Education, College of Life Sciences, Fujian Normal University, Fuzhou, China

**Keywords:** patent, PD-1, PD-L1, immunotherapy, clinical trial

## Abstract

Targeting PD-L1 and PD-1 interactions is a relatively new therapeutic strategy used to treat cancer. Inhibitors of PD-1/PD-L1 include peptides, small molecule chemical compounds, and antibodies. Several approved antibodies targeting PD-1 or PD-L1 have been patented with good curative effect in various cancer types in clinical practices. While the current antibody therapy is facing development bottleneck, some companies have tried to develop PD-L1 companion tests to select patients with better diagnosis potential. Meanwhile, many companies have recently synthesized small molecule inhibitors of PD-1/PD-L1 interactions and focused on searching for novel biomarker to predict the efficacy of anti-PD-1/PD-L1 drugs. This review summarized clinical studies and patent applications related to PD-1/PD-L1 targeted therapy and also discussed progress in inhibitors of PD-1/PD-L1.

## Introduction

**Figure 1:**
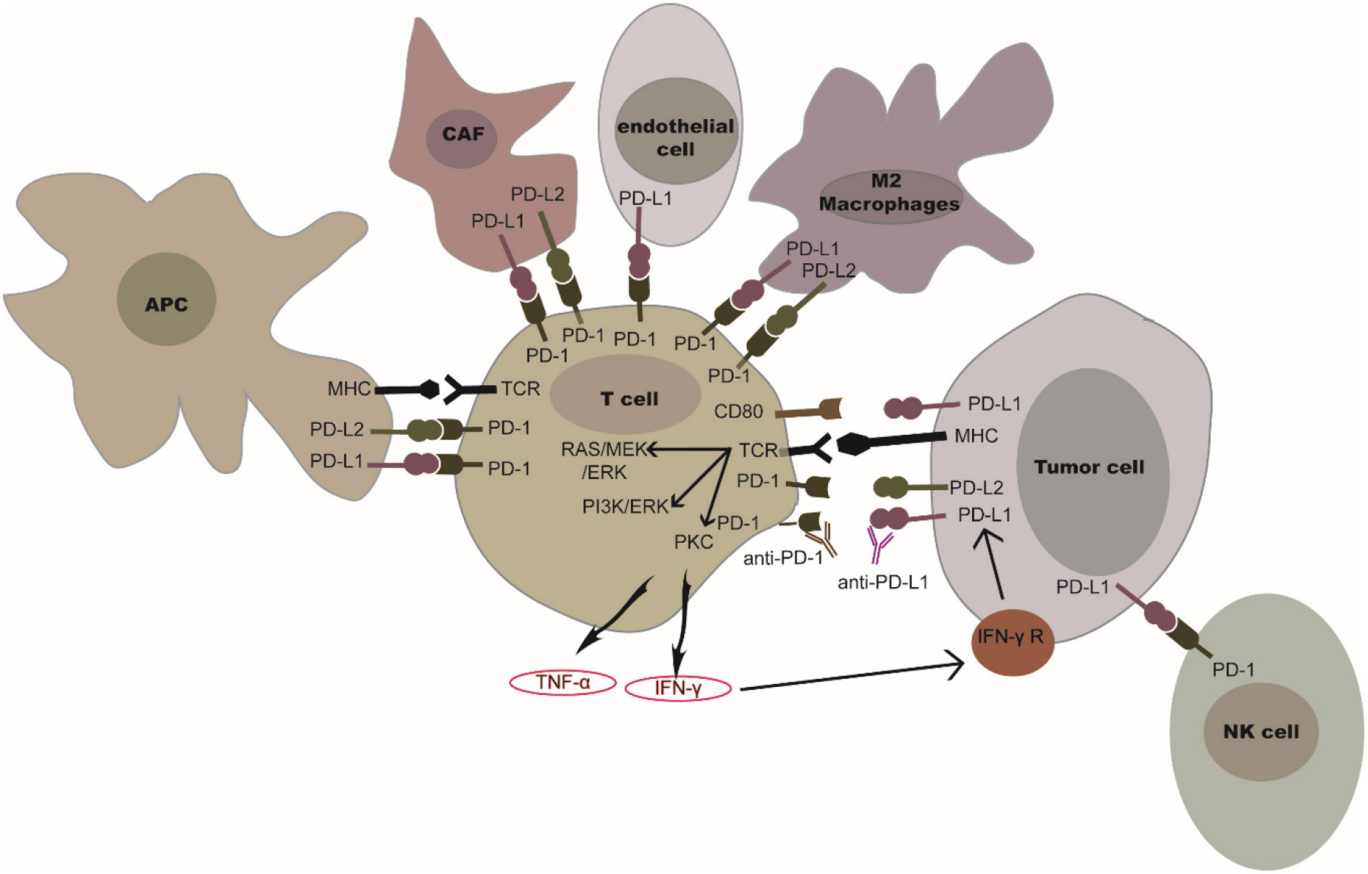
PD-1/PD-L1 or PD-1/PD-L2 in the tumor microenvironment. PD-1 is expressed on T-cells and NK cells. PD-L1 is expressed in tumor cells, antigen presenting cells, cancer associated fibroblasts, and in several immune cells (myeloid cells, endothelial cells, M2 macrophages). The binding of PD-L1 or PD-L2 to PD-1 could inhibit the functioning of T-cells and NK cells. IFN-γ secreted by activated T-cells mediates the up-regulation of tumor PD-L1. The blockade of PD-1/PD-L1 or PD-1/PD-L2 interaction by PD-1 or PD-L1 inhibitors could restore T-cell or NK cell activation.

Programmed cell death protein 1, also referred to as cluster of differentiation 279 (CD279), is a surface protein that can regulate the immune system by inhibiting T-cell activity. PD-1 is constitutively expressed on activated T-cells, B cells, natural killer (NK) cells, macrophages, and dendritic cells (DCs) (1). Programmed death-ligand 1 (PD-L1), also referred to as B7-H1 or CD274, is constitutively expressed on antigen-presenting cells, lymphoid, endothelial, and epithelial cells (2). Interferon gamma (IFN-γ) and tumor necrosis factor (TNF-α) secreted by activated T-cells can also induce PD-L1 expression on tumor cells and antigen-presenting cells (APCs) (3). Figure 1 shows that naïve T-cells are activated through binding between T cell receptors (TCR) and the peptide-MHC complex presented by (APC); T-cell activation can lead to transient upregulation of PD-1, which is the receptor of PD-L1. Binding between PD-1 and PD-L1 negatively regulates downstream signaling mediated by co-activation of TCR and CD28 (4). When PD-L1 interacts with PD-1, the immunoreceptor tyrosine-based inhibitory motifs (ITIM) and immunoreceptor tyrosine-based switch motifs (ITSM), which are on the intracellular domain of PD-1, can be phosphorylated. The Src homology 2 domain-containing protein tyrosine phosphatase 1 (SHP-1) and Src homology 2 domain-containing protein tyrosine phosphatase 1 (SHP-2) are then recruited and bind to ITIM to further inhibit the signaling downstream of the TCR (5). After inhibiting the TCR-mediated signaling pathway, PD-1 prevents the activation of the pathway mediated by PI3K/Akt or Ras/MEK/Erk. This further inhibits the function of CD8+ T-cells (6). Programmed cell death 1 ligand 2 (also known as PD-L2, B7-DC), which is the second ligand of PD-1, is expressed on tumor cells, APCs, cancer associated fibroblasts, and macrophages (7–9). PD-L2 plays an inhibitory role on the functioning of T-cells, which is similar to that of PD-L1. Meanwhile, PD-L1 also interacts with the surface protein CD80 (B7-1) expressed on activated T-cells. Interacting with PD-L1, CD80 could induce increased expression of Bim, which contributes to the apoptosis of CD8+ T-cells (10). As a result, the PD-1/PD-L1 signaling pathway promotes tumor cells escaping immune surveillance by inhibiting cell survival and activation of T-cells.

**Figure 2:**
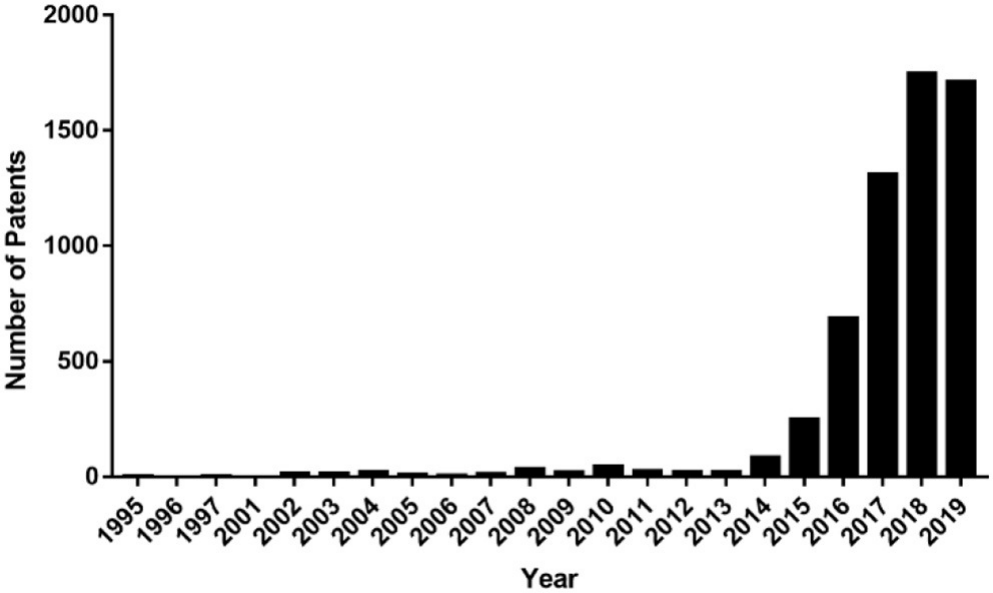
Numbers of international patent applications published per year containing the word “PD-1” or “PD-L1” in the title, claim, or abstract.

Targeting PD-L1 and PD-1 interactions is a novel therapeutic strategy used for cancer treatment. Antibodies targeting PD-1 or PD-L1 have marked a breakthrough in cancer immunotherapy and have become a hot topic in cancer therapy. Many companies have therefore begun studies on cancer immunotherapy and applied a series of related patents and patent applications in this field. To date, there have been about 5,000 patents published, and the number of patents continues to increase (Figure 2). In this review, we demonstrate the development of PD-1/PD-L1 directed immunotherapy and progress in inhibitors disrupting PD-1/PD-L1 binding. Moreover, patents or patent applications related to PD-1/PD-L1 signaling pathway and its inhibitors will also be discussed in this review, which will provide an update on PD-1/PD-L1 targeted cancer therapy.

## Historical Overview of Relevant Patents of PD-1/PD-L1 Targeting Cancer Immunotherapy

The PD-1 protein was discovered by Tasuku Honjo in 1992, and he was awarded the Nobel Prize in physiology and medicine in 2018. The patent published in 1995 by Honjo firstly proposed the sequence of PD-1 protein and gene encoded PD-1 (11). Honjo's discovery also showed that PD-1 is a protein that negatively regulates the immune system (12). Later, Gordon Freeman identified B7–4 as one of the ligands to PD-1 (13). Meanwhile, Dr. Lieping Chen and his team independently discovered B7-H1. The sequence of B7-H1 protein and gene encoded B7-H1 was published in 1999 by Dong et al. (14). However, they did not mention the correlation between B7-H1 and PD-1. Based on his own findings of B7-H1, Chen et al. applied a series of patents related to B7-H1 protein. Meanwhile, in 2000, Freeman et al. published a paper mentioning that B7-4 was renamed to PD-L1 and is the same as B7-H1 protein discovered by Freeman et al. (15). Freeman also mentioned that PD-L1 is one of the members of the CD28/B7 immunoglobulin superfamily that could inhibit the T-cell function through PD-1/PD-L1 interactions (15). Table 1 shows patents and patent applications for the finding of PD-1 and PD-L1 proteins and the development of PD-1/PD-L1 blockade therapy.

**Table 1:** Patents and patent applications naming Honjo, Freeman, and Dr. Chen as inventors that are related to PD-1 and PD-L1.

**Patent number**	**Inventors**	**Details**
US5698520A (11)	Honjo et al.	The sequence of nucleic acid and amino acid of PD-1
US7563869B2 (16)		The antibodies specifically binding to human PD-1 and the use of these antibodies.
US7038013B2 (17)	Freeman et al.	The nucleic acid sequence and amino acid sequence of PD-1 polypeptide and anti-B7-4 antibodies.
US7101550B2 (18)		PD-1 was recognized as a receptor for B7-4.
US8652465B2 (19)		A method of reducing viral titer by an anti PD-L1 antibody
US6808710B1 (20)		A method for down modulating an immune response by PD-1 antibody
US9062112B2 (21)	Chen et al.	The nucleic acid sequence can encode a B7-H1 polypeptide
US8981063B2 (22)		An isolated antibody that specifically binds to B7-H1
US7892540B2 (23)		A method for treating cancer with B7-H1 antibody

Honjo's studies suggested that suppression of the PD-1 protein could be effective in cancer treatment (12). Studies have shown that expression of PD-1 and PD-L1 was enhanced in cancer cells and was related to defective immune responses (24). These studies suggested that two immune checkpoint molecules may be important therapeutic targets for cancer and infectious disease treatment. Thus, the blockade of PD-1/PD-L1 interactions using inhibitors may be a novel and effective strategy for immunotherapy. Additionally, a previous study showed that blockade of the PD-1/PD-L1 pathway using PD-L1 antibody could inhibit T-cell apoptosis (25). This study also showed that PD-L1 antibody affected the survival of tumor cells *in vivo* (25). These results proved that PD-L1 antibodies can enhance T-cell growth to further inhibit tumor growth—this suggests that inhibition of the PD-1/PD-L1 interaction could be a new method of cancer treatment.

Honjo cooperated with Ono Pharmaceutical Co. and Medarex to develop an anti-cancer medication targeting PD-1, named nivolumab. Two studies of nivolumab conducted in Phase III trials showed impressive efficacy for this antibody in advanced melanoma (26, 27). The results of a phase III trial showed that the overall survival rate at 1 year was significantly different between the nivolumab group (72.9%) and dacarbazine group (42.1%) of previously untreated patients who had advanced melanoma without a BRAF mutation (26). In addition, nivolumab showed higher response rates and lower toxicity rates than ipilimumab and chemotherapy (27). Following the results of these two clinical trials, the Food and Drug Administration (FDA) approved nivolumab for the treatment of advanced melanoma in 2014. The discovery of the PD-1/PD-L1 signaling pathway attracted researchers' attention on developing antibodies against this pathway. The PD-1 protein has led to breakthroughs in cancer immunotherapies in the past decades. Many companies have filed patents related to antibodies during these past 20 years. Table 2 shows the core patents related to FDA-approved antibodies while Table 3 shows patents related to antibodies.

**Table 2:** The key patents related to FDA-approved anti-PD-1/L1 antibodies.

**Target**	**Drug**	**Company**	**Patent number**	**Inventor**	**Antibody class**
PD-1	Nivolumab	BMS/Ono	US7595048	Honjo et al. (28)	IgG4
	Pembrolizumab	Merck&Co	US8952136	Carven et al. (29)	IgG4
PD-L1	Avelumab	MerckSerono	US2014341917	Nastri et al. (30)	IgG1
	Atezolizumab	Roche	US8217149	Irving et al. (31)	IgG1
	Durvalumab	AstraZeneca	US8779108	Queva et al. (32)	IgG1

**Table 3:** The patents related to currently developed anti-PD-1/L1 antibodies.

**Target**	**Drug**	**Company**	**Patent number**	**Inventor**	**Antibody class**
PD-1	Spartalizumab (PDR-001)	Novartis	US9683048B2	Freeman et al. (33)	IgG4κ
	Cemiplimab (Libtayo)	Regeneron Pharmaceuticals	US20150203579	Papadopoulos et al. (34)	IgG4
	Camrelizumab (SHR-1210)	Incyte Biosciences and Jiangsu Hengrui Medicine	US20160376367A1	Yuan et al. (35)	IgG4
	Tislelizumab (BGB-A317)	BeiGene	US8735553B1	Li et al. (36)	IgG4
	Dostarlimab (TSR-042)	Tesaro/AnaptysBio	US9815897B2	King et al. (37)	IgG4
	MEDI-0680 (AMP-514)	MedImmune LLC	US8609089B2	Langermann et al. (38)	IgG4
	SSI-361	Lyvgen	US20180346569A1	Wang et al. (39)	IgG4
	AMP-224	Amplimmune Inc	US20130017199	Langermann et al. (40)	PD-L2 IgG2a fusion protein
PD-L1	CX-072	CytomX	US20160311903A1	West et al. (41)	protease activatable prodrug
	BMS-936559 (MDX 1105)	Medarex Inc	US7943743	Korman et al. (42)	IgG4
	KN035	Jiangsu Alphamab Biopharmaceuticals Co., Ltd.	US20180327494A1	Xu et al. (43)	fusion protein of humanized anti-PD-L1 single domain antibody and human IgG1 Fc

## Structure Analysis of Antibodies Targeting PD-1 and PD-L1

Several structures and classes of antibodies inhibiting the PD-1/PD-L1 interaction have been published recently. Most of these anti-PD-1 antibodies are fully human immunoglobulin G4 (IgG4) antibodies with the S228P mutation, including nivolumab, pembrolizumab, cemiplimab, dostarlimab, MEDI-0680, and SSI-361. These antibodies have similar binding properties to the natural IgG4, which reduce ADCC function and eliminate CDC function, but they still retain function in binding to FcγRI and FcγRIIb. Spartalizumab is a humanized IgG4κ monoclonal antibody with S228P mutations and K447 deletion (44). Tislelizumab was generated via the introduction of several mutations (including S228P, E233P, F234V, L235A, D265A, and R409K) in IgG4 antibodies (45). AMP-224 is an anti-PD-1 recombinant fusion protein that contains the extracellular domain of PD-L2 and Fc domain of human IgG1 (46).

Moreover, the crystal structures of PD-1/Anti-PD-1 antibodies have also been explored. The N-terminal extension, BC-loops, and FG-loops are crucial for binding of nivolumab and PD-1. The VL chain of nivolumab and PD-L1 residues shared an overlapping binding surface on the FG loop (47). The C'D loop of PD-1 mainly contributes to the interaction with pembrolizumab (48). Anti-PD-1 antibodies inhibit the PD-1/PD-L1 interaction by competing with PD-L1 while binding to PD-1. The epitopes of these antibodies directly occupy the partial binding site of the PD-L1 protein. In addition, the binding of PD-1 and its antibodies induces optimal conformational changes in the PD-1 protein, which blocks PD-1/PD-L1 interactions, because PD-1 also interacts with PD-L1 in distinct conformations. Tislelizumab interacts with an IgV-like domain of PD-1 and is different from pembrolizumab and nivolumab, as shown by its unique binding epitopes, including Gln75, Thr76, Asp77, and Arg86 (45). Although SHR-1210 was reported to have unspecific interactions with some human receptors driving angiogenesis, the optimization of complementary determining region (CDR) domains successfully eliminated off-target binding (49). Meanwhile, the binding properties of SHR-1210 have not been reported.

Unlike anti-PD-1 antibodies, three approved anti-PD-L1 antibodies include human IgG1 antibodies. Atezolizumab and durvalumab are antibodies of eliminated FcγR-binding and effector functions while avelumab was designed to retain intact Fc functions (50). BMS-936559 is differentiated from three approved PD-L1 antibodies and is an IgG4 mAb with S228P mutations (50). KN035 is a fusion protein containing a single domain of the humanized anti-PD-L1 antibody and the Fc of an IgG1 (51). CX-072 is a human PD-L1 specific protease-activatable antibody prodrug. CX-072 was designed by linking the masking peptide links to the targeted antibody (52).

Recently, the crystal structures of the PD-L1/avelumab complex revealed that avelumab/atezolizumab/BMS-936559 binds to the IgV domain of PD-L1 through its heavy chain (VH) and light chain (VL). These are dominated by the VH chain (53). A comparison of the PD-L1/antibody and human PD-1/PD-L1 complexes demonstrates that antibodies directly occupy the partial binding site of the PD-1 protein. In contrast, the PD-L1/durvalumab Fab complex demonstrated that the binding sites of the antibody are in the N-terminal region of the PD-L1 protein (53). The KN035/PD-L1 complex showed a different pattern. The paratope of KN035 is limited to only two complementary determining regions (CDRs)—one of which contributes to binding with high-affinity (54). This narrow binding area provides an opportunity for rationally designing peptides or small-molecule inhibitors that imitate the nanobody/PD-L1 interface.

## Clinical Application of PD-1/PD-L1 Targeting Cancer Immunotherapy

**Figure 3:**
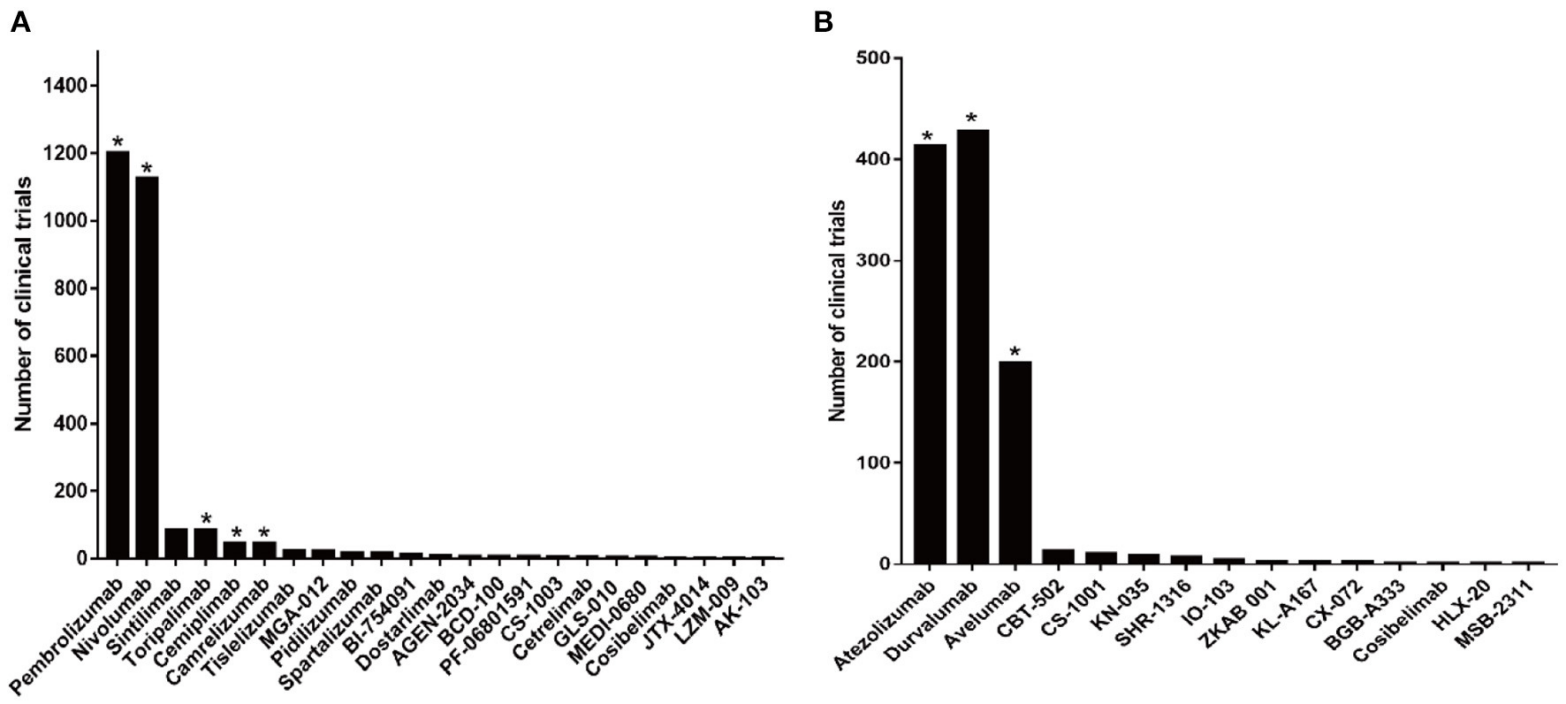
Clinical trials related to anti-PD-1/PD-L1 antibodies. **(A)** Numbers of clinical trials of anti-PD-1 antibodies. **(B)** Numbers of clinical trials of anti-PD-L1 antibodies. Antibodies that obtained approval for cancer therapy are indicated by an asterisk.

There have been more than 2,000 clinical trials of anti-PD-1 antibodies and over 1,000 clinical trials of anti-PD-L1 antibodies (Figure 3). Based on the data from several clinical trials, some of these drugs have been approved by the FDA, the National Medical Products Administration (NMPA), and the European Medicines Agency (EMA) for use in the treatment of various cancers. Nivolumab and pembrolizumab, two anti-PD-1 antibodies, obtained approval for cancer therapy in 2014. After that, more PD-1 and PD-L1 drugs got FDA approval following positive results from clinical trials. There are currently several FDA-approved antibodies, including nivolumab (trade name: Opdivo), pembrolizumab (trade name: Keytruda), cemiplimab (trade name: Libtayo), atezolizumab (trade name: Tecentriq), durvalumab (trade name: Imfinzi), and avelumab (trade name: Bavencio) (Table 4) (50). In addition, camrelizumab and toripalimab were approved by NMPA for marketing.

**Table 4:** Drugs approved by FDA, NMPA, and EMA for cancer immunotherapy.

**Target**	**Drug**	**Indication**	**Related clinical trials no**	**Phase**	**Remark**
PD-1	Nivolumab	Deficiency mismatch repair (dMMR) or MSI-H metastatic colorectal cancer	NCT02060188 (55)	II	First line
		Melanoma	NCT01721746 (56)	III	First line
		Metastatic squamous Non-small-cell lung carcinoma (NSCLC)	NCT01673867 (57)	III	First line
		Metastatic non-squamousNSCLC	NCT01673867 (58)	III	Second line
		Locally advanced or metastatic urothelial carcinoma (UC)	NCT02387996 (59)	II	Second line
		Advanced Renal cell carcinoma	NCT01668784 (60)	III	Second line
		Hematologic malignancy	NCT01592370 (61); NCT02181738 (62)	I; II	Second line
		Advanced hepatocellular Carcinoma	NCT01658878 (63)	I&II	First line
		Recurrent/Metastatic Head and neck squamous cell carcinoma (HNSCC)	NCT02105636 (64)	III	First line
	Pembrolizumab	Advanced or unresectable melanoma	NCT01295827 (65, 66)	I	First line
		Advanced or metastatic PD-L1-positive NSCLC	NCT01295827 (67)	I	First line
		Locally advanced or metastatic UC	NCT02335424 (68); NCT02256436 (69)	II; III	First line
		Recurrent or metastatic HNSCC	NCT01848834 (70)	Ib	First line
		Hematologic malignancy	NCT02181738 (62)	II	third line therapy or greater
		Microsatellite instability or mismatch repair deficient cancers	NCT01876511 (71)	II	Second line
		Advanced gastroesophageal Cancer	NCT02335411 (72)	II	First line
		Metastatic Cervical Cancer	NCT02628067 (73)	II	First line
		Locally advanced or metastatic, esophagus squamous cell carcinoma (ESCC)	NCT02559687, NCT02564263	II	First line
	Cemiplimab	Advanced cutaneous squamous cell carcinoma (CSCC)	NCT02383212, NCT02760498	I&II	First line
	Camrelizumab	Classical Hodgkin lymphoma (cHL)	CTR20170500/NCT03155425/ SHR-1210-II-204	II	Second-line therapy or greater
	Toripalimab	Malignant melanoma	NCT03013101	II	First line
PD-L1	Avelumab	Locally advanced or metastatic UC	NCT01772004 (74)	Ib	Second line
		Metastatic Merkel cell carcinoma	NCT02155647 (75)	II	Second line
	Atezolizumab	Previously treated metastatic NSCLC	NCT01903993 (76); NCT02008227 (77)	II; III	Second line
		Locally advanced and metastatic UC	NCT02108652 (78)	II	First line
	Durvalumab	Locally advanced, unresectable NSCLC	NCT02125461 (79)	III	First or second line
		Locally advanced or metastatic UC	NCT01693562 (80)	I&II	Second line

Clinical trials of most antibodies have just started, and the results require further updating. Table 5 summarizes several clinical trials of anti-PD-1/PD-L1 antibodies that are currently being developed with the latest data. The data from clinical trials revealed that newly developed antibodies also showed a durable response. Table 5 also demonstrates that anti-PD-1/PD-L1 antibodies can cause treatment-related adverse effects (TRAEs) and immune-related adverse effects (IRAEs). In some patients, these AEs led to treatment discontinuation and treatment interruption. The Objective response rate (ORR) is 47% among the 75 patients with metastatic CSCC who received cemiplimab-rwlc. Complete response was achieved in 4% of patients (81). Among patients with relapsed/refractory cHL (NCT02961101 and NCT03250962), the response duration rate at 6 months was 76% in patients treated with camrelizumab monotherapy (*n* = 19) compared to 100% in those treated with decitabine plus camrelizumab (*n* = 42) (95). Among the 127 patients with advanced melanoma (NCT03013101), the ORR is 17.3% in overall population after treatment with toripalimab. The disease control rate (DCR) was 57.5% and median progression free survival (PFS) was 3.6 months (96). Based on the clinical results shown above, cemiplimab, camrelizumab, and toripalimab were approved for clinical use.

**Table 5:** Results of clinical evaluation of selected anti-PD-1 or anti-PD-L1 antibodies.

**Target**	**Antibody**	**Pivotal indications**	**Most advanced phase**	**Most recent result**	**Most common adverse effects (AEs)**
PD-1	Cemiplimab (Libtayo)	Squamous cell cancer	Phase IIII	Metastatic CSCC (81): ORR: 47% (95% CI, 34–61); Median follow-up months: 7.9	The most common AEs were diarrhea (27%). 4 patients (7%) had AEs leading to discontinuation.
	Pidilizumab (CT-011)	Relapsed Follicular Lymphoma	Phase II	Pidilizumab + rituximab (82): ORR: 66% Complete response (CR): 52% partial response (PR): 14% Median follow-up months: 18.8 (95% CI: 14.7 months to not reached)	Anemia (14/29), Fatigue (13/29).
	Spartalizumab (PDR-001)	BRAF V600–mutant unresectable or metastatic melanoma.	Phase III	Spartalizumab (S) + dabrafenib (D) + trametinib (T) (83): ORR: 75% CR: 33% Median follow-up months: 12 (95% CI, 47–79%)	27 (75%) had grade ≥ 3 AEs. 6 patients (17%) had AEs leading to discontinuation.
	Camrelizumab (SHR-1210)	Nasopharyngeal cancer	Phase III	Camrelizumab monotherapy (84): ORR: 34%; 95% CI 24–44 Median follow-up months: 9.9	15 (16%) patients had AEs of grade 3 or 4
	Tislelizumab (BGB-A317)	Nasopharyngeal cancer	Phase III	Tislelizumab (85): PR: 15% Stable disease (SD): 45% Median follow-up months: 5.5	Hypothyroidism (3/20). No AEs led to discontinuation.
	Toripalimab (TAB001, JS001)	Advanced melanoma	Phase III	Toripalimab (86): ORR: 20.7% PR: 19.8% SD: 39.6%	Proteinuria (25%), ALT increase (25%)
	Dostarlimab (TSR-042)	Advanced NSCLC and microsatellite instability-high (MSI-H) Endometrial cancer (EC)	Phase III	TSR-042 (87): NSCLC group: PR: 33.3% SD: 28.6% MSI-H EC group: PR: 36.4% SD: 18.2%	Diarrhea (22.4%) Nausea (22.4%)
	AGEN-2034	Cervical cancer; Solid tumors	Phase I&II	AGEN2034 (88): PR: 12% SD: 52%	2 patients (6%) had AEs leading to discontinuation.
	Sintilimab (IBI-308)	Relapsed/refractory classical Hodgkin's Lymphoma (HL)	Phase III	Sintilimab (89): ORR: 80.4%; 95% CI 70.9–88.0 Median follow-up: 10.5 (9.2–1) months; Six-month PFS: 77.6% (66.6–85.4)	93% patients had treatment-related adverse events. The most common AEs were pyrexia (3%).
	BCD-100	Malignant melanoma	Phase III	BCD-100 1 mg/kg (90): ORR: 34% CR: 6.7% PR: 27.1% DCR: 68%. BCD-100 3 mg/kg: ORR: 29% CR: 3.6% PR: 25.4% DCR: 55%.	BCD-100 1 mg/kg: TRAEs (48%); IRAEs (29%). BCD-100 3 mg/kg: TRAEs (48%); IRAEs (30%).
	GLS-010	Hodgkin's disease	Phase II	GLS-010 (91): ORR: 88.3% CR: 23.5% PR: 64.7% SD: 5.9%	The most common treatment related AEs were Neutrophil (31.25%),
PD-L1	CX-072	Solid tumors	Phase II	CX-072 (92): PR: 8% SD: 43% PD: 47%	2 patients had AEs leading to discontinuation.
	WBP-3155 (CS1001)	Advanced solid tumors or lymphomas	Phase III	CS1001 (93): PR: 24% SD: 28%	Anemia (48%). 2 patients had AEs leading to discontinuation.
	Cosibelimab (CK-301)	Cancer	Phase I	Cosibelimab (94): NSCLC group: ORR: 42% DCR: 83% CSCC group: ORR: 43%, DCR: 86%. In melanoma and HL group: ORR: 14% DCR: 71% Colorectal cancer group: ORR: 10% DCR: 60%	Most common AEs were rash (14%)

## The Current Optimization of Anti-PD-1/PD-L1 Treatment Strategy

Several clinical trials using antibodies targeting the interaction of PD-1 and PD-L1 for cancer treatment have shown promising abilities in prolonging survival, but not all patients respond to PD-1/PD-L1 inhibitors (97). In addition, clinical results have also shown that anti-PD-1 or anti-PD-L1 treatment caused TRAEs and IRAEs, although anti-PD-1/PD-L1 drugs have shown lower toxicity than standard chemotherapy (98). Most seriously, AEs caused by these antibodies sometimes could lead to treatment discontinuation and treatment interruption (98). Due to the limited success and disadvantages of anti-PD-1/PD-L1 antibodies, effective strategies are needed to improve the efficacy of PD-1/PD-L1 targeted immunotherapy. Detecting PD-L1 expression in tumor cells and tumor infiltrated T-cells would be useful for targeting patients with a big likelihood of responding to PD-1/PD-L1 treatment. Meanwhile, it is also crucial to search for potential biomarkers that could selectively reflect the efficacy and feasibility of anti-PD1/PD-L1 therapy. Furthermore, small molecule inhibitors targeting PD-1 and PD-L1 are emerging as their potential advantages are realized vs. monoclonal antibodies.

### The Application of PD-L1 Immunohistochemistry (IHC) Assays

Some clinical trials have shown that more than half of patients had no response to anti-PD-1 drugs, and some responders even experience tumor relapse within 2 years after treatment of anti-PD-1 drugs (26, 99). Studies suggest that clinical efficacy of PD-1/PD-L1 targeted immunotherapies may be predicted by PD-L1 expression on tumor cells and tumor-infiltrating immune cells (100). Developing PD-L1 IHC test compounds have begun to attract scientists' attention during the past 5 years. Several companies have designed commercially available PD-L1 IHC tests, including 22C3, 28-8, SP263, SP142, E1L3N, and 73-10 assays. Merck developed a PD-L1 IHC test using 22C3 antibody and also applied for a patent (US9709568B2), which claimed the use of the 22C3 antibody for diagnostic purposes (101). In addition, BMS designed a different companion assay for PD-L1 expression using its 28-8 antibody and had a patent application (WO2013173223A1) that described a method of detecting PD-L1 expression using the clone 28-8 antibody (102). The SP142 assay was developed by Ventana and was described in patent application WO2015181343A2 (103).

These PD-L1 IHC assays are currently being tested in clinical trials, and some of them have been approved by the FDA as companion diagnostics for PD-1/PD-L1 targeted immunotherapies. Table 6 shows that PD-L1 expression was first reported to be associated with higher response rates to pembrolizumab/atezolizumab and was approved by the FDA to guide the selection of patients for anti-PD-1/PD-L1 treatment. For example, the DAKO 22C3 IHC assay is approved for use as a companion diagnostic with pembrolizumab immunotherapy in NSCLC, gastric cancer, cervical cancer, HNSCC, and ESCC (68, 104–108). In addition, the Ventana PD-L1 (SP142) assay has also been approved as a companion diagnostics test for atezolizumab in UC and TNBC (109, 110). IHC 28–8 and SP263 (nivolumab and durvalumab, respectively) are complementary diagnostics and have not been approved by the FDA. Recent studies (e.g., shown in the meta-analysis) have also confirmed that efficacy of PD-1/PD-L1 inhibitors was more sensitive in PD-L1 positive patients compared to negative groups (111). Each PD-L1 IHC assay, performed in different IHC staining platforms, is independently developed for a specific anti-PD-1 or anti-PD-L1 drug. As is shown in Table 7, differences between six commonly used PD-L1 IHC assay were shown by detection system, staining platform, and antibody epitope. Thus, each assay potentially displayed different staining sensitivities. Different PD-L1 IHC assays and different PD-L1 tumor expression cut-off points are used in clinical trials, which raises concerns about whether the tests can be used interchangeably. The Blueprint PD-L1 IHC Assay Comparison Project was founded to enable a better understanding of the similarities and differences between these four PD-L1 IHC systems. This project is an industrial-academic partnership seeking to harmonize IHC PD-L1 testing. The result from phase I of the Blueprint project showed that the 22C3, 28-8, and SP263 assays displayed comparable sensitivity and the SP142 assay showed significantly less sensitivity (112). The phase I of the Blueprint project detect PD-L1 expression on TCs using four PD-L1 IHC assays performed in different staining platforms, and the result of staining was evaluated independently by three pathologists (112). Phase 2 of the Blueprint project compares 73-10 assay with four other PD-L1 IHC assay (including 22C3, 28-8, SP263). The results from phase 2 showed highly comparable sensitivity between 22C3, 28-8, and SP263 assays, less sensitivity with SP142 assay, and higher sensitivity with 73-10 assay when detecting PD-L1 expression on TC (113). The high concordance was observed between scorings by glass slide and scorings by digital image (113). Most importantly, a recent study has investigated the cause of distinct immunohistochemical staining generated by SP142 assay. The results suggested that discordances are more likely caused by differences of staining platform rather than antibody epitope (114).

**Table 6:** Summary of studies on the PD-L1 IHC assay.

**Study information**	**Population**	**Cut-off value of PD-L1 expression**	**Response**
PD-L1 IHC assay: DAKO 22C3 IHC assay Drug: Pembrolizumab	NSCLC (104)	Tumor proportion score (TPS) > 1%	TPS<1%: 8.3% (ORR) 1%≤TPS≤49%: 17.3% (ORR) TPS>50%: 51.9% (ORR)
	Gastric or gastroesophageal junction adenocarcinoma (105)	Combined proportion score (CPS)≥1	CPS≥1: 16% (ORR) CPS<1: 6% (ORR)
	Cervical cancer (106)	CPS≥1	CPS≥1: 14.3% (ORR) CPS<1: 0 (ORR)
	UC (68)	CPS>10	CPS>10: 39% (ORR) 1% ≤ CPS ≤ 10%: 20% (ORR) CPS<1: 11% (ORR)
	HNSCC (107)	CPS≥1	Median overall survival: Pembrolizumab vs. cetuximab plus chemotherapy: 12.3:10.3 (HR 0.78; 95% CI: 0.64, 0.96; *p* = 0.0086)
	ESCC (108)	CPS≥10	Median OS: Pembrolizumab vs. chemotherapy: 10.3:6.7 (HR 0.64; 95% CI: 0.46, 0.90); ORR: Pembrolizumab vs. chemotherapy: 22%: 7%
PD-L1 IHC assay: Ventana SP142 IHC assay Drug: Atezolizumab	UC (109)	PD-L1 tumor infiltrating immune cell (IC) expression ≥5%	IC≥5%: 26% (ORR) IC<5%: 9.5% (ORR)
	Triple-negative breast cancer (TNBC) (110)	PD-L1 IC expression ≥1%	IC≥1%: 12% (ORR); 15% (DCR) IC<1%: 0% (ORR); 5% (DCR)

**Table 7:** The comparison of commonly used PD-L1 IHC assay.

**Antibody clone**	**Manufacturer**	**Detection systems**	**Staining platform**	**Species**	**Heat-induced epitope retrieval**	**Binding sites of antibody**
22C3,28-8,73-10	Dako	EnVision FLEX visualization system	Dako Autostainer Link 48	Rabbit	EnVision FLEX	extracellular domain of PD-L1
SP142, SP263	Ventana/Roche	OptiView detection kit	Ventana BenchMark ULTRA	Rabbit	CC1 Cell conditioning	the cytoplasmic domain at the extreme C-terminus of PD-L1
E1L3N	Cell Signaling Technology	Laboratory detection system	Laboratory detection system	Rabbit	Laboratory detection system	cytoplasmic domain of PD-L1

### The Current Potential Biomarkers Used to Evaluate the Feasibility of Anti-PD-1/PD-L1 Therapy

PD-1/PD-L1 inhibitors represent a breakthrough in cancer therapy. However, the response rates of PD-1/PD-L1 inhibitors in patients is, overall, unsatisfactory and results in limited applications in clinical practice. Therefore, searching for biomarkers predicting the efficacy of PD-1/PD-L1 inhibitors is crucial for patient selection. There are several biomarkers associated with the response to anti-PD-1/anti-PD-L1 therapy (Table 8) including PD-L1 expression, lactate dehydrogenase (LDH), mismatch-repair (MMR) deficiency, gene alteration, tumor mutational burden, etc. A clinical study conducted by Diem showed that patients with an elevated baseline LDH showed a significantly shorter OS (*P* = 0.0292) and lower response rate compared with patients with normal LDH at baseline and during treatment. This suggests that LDH could predict early response or progression in advanced melanoma patients with anti-PD-1 therapy (115, 116). In addition, patients who achieved clinical benefit after treatment of anti–PD-1 therapy were detected with a higher percentage of Bim^+^PD-1^+^CD8^+^ T-cells in the peripheral blood (117). The levels of Bim in PD-1^+^CD11a^hi^CD8^+^ T-cells (also indicated tumor reactive T cell) could be a predictive factor of clinical benefit in patients with metastatic melanoma treated with anti–PD-1 therapy (117). High pretreatment lymphocyte count (LC) and relative eosinophil count (REC) were associated with improved overall survival of melanoma patients with pembrolizumab treatment (118). Patients with T-cells expressing SRY-Box 2 (SOX-2) experienced disease regression following the treatment of nivolumab, suggesting that SOX-2 is associated with a clinical response upon immunotherapy with anti-PD-1 monoclonal antibodies (119). A retrospective study showed that the median PFS of patients with a neutrophil-lymphocyte ratio (NLR) of ≥3 was shorter than that in patients with a NLR of < 3 (2.0 vs. 5.3 months, *p* = 0.00515) at 4 weeks after treatment (120). The clinical data suggested that the NLR ratio might be an indicator of a poor prognosis in patients with advanced NSCLC receiving nivolumab (120). Patients with a 1.5-fold increase in circulating soluble PD-L1 (sPD-L1) concentrations were more likely to achieve partial responses to anti–PD-1 antibodies after 5 months upon anti-PD-1 therapy. This shows the predictive effect of sPD-L1 on clinical response to anti-PD-1 therapy (121). Among 36 EGFR-mutated metastatic NSCLC patients, compared with patients detecting decreased levels of sPD-1, patients with an increased or stable sPD-1 level achieved longer PFS (*p* = 0.004) and OS (*p* = 0.002) after two cycles of nivolumab (122). In melanoma, the pre-treatment tumors in responding patients were detected with higher expressions of IFN-γ and IFN-γ-inducible genes, including indoleamine 2,3-dioxygenase 1 (IDO1) and C-X-C motif Chemokine Ligand 9 (CXCL9) (123). These associations were also found in NSCLC or renal cell carcinoma patients (123). In addition, genetic aberrations within tumors were also found to be associated with clinical efficacy in anti-PD-1/PD-L1 therapy. For example, among 155 patients, six patients with MDM2/MDM4 amplification and seven of eight patients with Epidermal Growth Factor Receptor (EGFR) alterations were found to have time-to-treatment failure (TTF) <2 months (124). Meanwhile, hyper-progressors harbored MDM2/4 amplifications or EGFR alterations (124). A retrospective analysis showed that EGFR-mutant and ALK-positive NSCLC patients receiving anti-PD-1/PD-L1 therapy showed lower ORR (*P* = 0.053) (125). Immunotherapeutic analysis and prospective observation suggested that patients harboring TP53 or KRAS mutations—especially co-mutations of TP53/KRAS—showed significantly better clinical responses to anti-PD-1 therapy (126). Among the 174 lung adenocarcinoma (LUAC) patients with KRAS mutations, patients harboring (Serine/Threonine Kinase 11) STK11 alterations showed lower ORR to PD-1 inhibitors vs. LUAC patients with mutant KRAS and wildtype STK11 (*P* < 0.001) (127). Another study evaluated the clinical efficacy of PD-1 inhibitors in patients with MMR-deficient tumors across 12 tumor types. ORR was achieved in 53% of patients, disease control was achieved in 77% of patients, and complete responses were achieved in 21% of patients (71). The MMR deficiency was defined by the presence of either MSI-H or by loss of MutL Homolog 1 (MLH1), MutS Homolog 2 (MSH2), MutS Homolog 6 (MSH6), or PMS1 Homolog 2 (PMS2) protein expression. Among the 35 patients with clear cell renal cell carcinoma (ccRCC), a clinical benefit was associated with loss-of-function mutations in the Polybromo 1 (PBRM1) gene (*p* = 0.012) after treatment of pembrolizumab and nivolumab (128). The presence of DNA damage response gene (DDR) alteration was associated with a higher response rate (*P* < 0.001) (129). The most commonly altered genes were ATM (*n* = 7), DNA Polymerase Epsilon (POLE) (*n* = 3), and BRCA2, ERCC2, FA Complementation Group A (FANCA), and MutS Homolog 6 (MSH6) (*n* = 2) (129). Gene variations that occur in at least 1% of the population used to be called polymorphism. Single nucleotide polymorphisms (SNPs) of tumor microenvironment-related genes (including *CCL2, NOS3, IL1RN, IL12B, CXCR3, and IL6R*) were significantly associated with ORR of patients treated with anti-PD-1/PD-L1 therapies (130). And safety of anti-PD-1/PD- L1 targeted therapies was significantly associated with gene SNPs including *UNG, IFNW1, CTLA4, PD-L1, and IFNL4 genes* (130). Besides that, rs17388568, which maps to a locus of IL2 gene and IL21 gene, was correlated with a higher response to anti-PD-1 targeting therapy (131). CD8, PD-1, and PD-L1 expression in the tumor and at the invasive margin significantly correlated with treatment outcome (*P* = 0.001) (132). Versus the progression group, the response group had significantly higher numbers of CD8^+^, PD-1^+^, and PD-L1^+^ cells (CD8, *P* = 0.0001; PD-1, *P* = 0.0002; PD-L1, *P* = 0.006) (132). Among HNSCC patients treated with pembrolizumab, PD-L2-positive patients showed higher ORR compared with PD-L2-negative patients (133). And longer PFS and OS were observed in PD-L2–positive patients (133).

**Table 8:** Current investigational biomarkers for PD-1/PD-L1 targeting therapy.

**Biomarkers**	**Population**	**Drug**	**End point result**	**References**
LDH	Melanoma	Ipilimumab Pembrolizumab	LDH level: Elevated group vs. Normal group: Median: 9.7 vs. not reached; 6-month OS: 60.8% vs. 81.6%; 12-month OS: 44.2% vs. 71.5%; *P* = 0.0292	(115)
	Melanoma	Pembrolizumab Nivolumab	LDH level: Elevated group: 22.3, 95% CI (17.1–28.1) Normal group 42.0, 95% CI (36.6–47.5)	(116)
Bim levels in circulating T cells	melanoma	Pembrolizumab	In patients with 4 cycles of anti–PD-1 therapy with clinical benefit, higher percentage of Bim^+^PD-1^+^CD8^+^ T cells in the peripheral blood was detected.	(117)
REC, LC	Melanoma	Pembrolizumab	High REC and absolute LC were negatively related with OS. *P* < 0.001	(118)
SOX-2 reactive T-cells	NSCLC	Nivolumab	Patients who responded to therapy (partial response, PR; *n* = 5) showed significantly greater immune response against SOX2 as compared non-responder (*p* = 0.02).	(119)
NLR	NSCLC	Nivolumab	NLR of <3 vs. NLR of ≥3: 2 weeks after treatment Median PFS: 5.3 vs. 2.1 months (*P* = 0.00528) 4 weeks after treatment Median PFS: 5.3 vs. 2.0 months (*P* = 0.00515)	(120)
sPD-L1	Melanoma	Pembrolizumab	Eight patients with ≥1.5-fold increases in sPD-L1^all^ after 5 months of treatment experienced partial responses (Fisher exact test *P* = 0.007), and four patients with ≥1.5-fold increases in sPD-L1^L^ after 5 months of treatment experienced partial responses (Fisher exact test, *P* = 0.103)	(121)
sPD-1	NSCLC	Nivolumab	After two cycles of nivolumab, an increased or stable sPD-1 level independently correlated with longer PFS (HR: 0.49, *p* = 0.004) and OS (HR: 0.39, *p* = 0.002).	(122)
IFN-γ,IDO1, CXCL9	Melanoma, NSCLC, RCC	Atezolizumab	Higher expression of IFN-γ and IDO1 as well as CXCL9 were detected in pretreatment tumors in responding patients. *P* = 0.024	(123)
Mutation of EGFR, MDM2, MDM4	Adenocarcinoma of lung Bladder carcinoma Breast cancer endometrial stromal sarcoma	Pembrolizumab Nivolumab Atezolizumab	Alteration of EGFR and MDM2/4 showed significance for correlation with TTF <2 months (*p* = 0.02).	(124)
ALK, EGFR	NSCLC	PD-1/PD-L1 inhibitors (Pembrolizumab, Nivolumab, Atezolizumab, Durvalumab, other)	Objective response (OR): EGFR-mutant or ALK-positive patients: 1/28 (3.6%); EGFR wild-type and ALK-negative/unknown patients: 7/30 (23.3) *P* = 0.053	(125)
KRAS/TP53	NSCLC	Pembrolizumab Nivolumab	Median PFS: TP53-mutant vs. KRAS-mutant vs. wild-type: 14.5 vs. 14.7 vs. 3.5 months; *P* = 0.012	(126)
STK11	KRAS mutant -LUAC	PD-1/PD-L1 inhibitors (Pembrolizumab, Nivolumab, Atezolizumab)	KRAS-mutant LUAC: Objective response rates: KL vs. KP vs. K-only: 7.4% vs. 35.7 vs. 28.6%, *P* < 0.001; Patients treated with nivolumab: KL vs. KP vs. K-only: 0 vs. 57.1 vs. 18.2%; *P* = 0.047.	(127)
MMR deficiency	12 different tumor types	Pembrolizumab	Objective radiographic responses were noted in 53% of patients (95% CI, 42–64%). Disease control was achieved in 77% of patients (95% CI, 66–85%). complete radiographic response was achieved in 21%.	(71)
PBRM1	ccRCC	Nivolumab Atezolizumab	PBRM1 were enriched in tumors from patients in the CB vs. NCB group (9/11 vs. 3/13; Fisher's exact *p* = 0.012, *q* = 0.086)	(128)
DDR gene	Advanced urothelial cancers	Nivolumab Atezolizumab	ORR: known or likely deleterious DDR alterations vs. unknown significant DDR alterations vs. wildtype DDR: 67.9 vs. 80 vs. 19%, *P* < 0.001	(129)
Single nucleotide polymorphisms (SNPs) of tumor microenvironment-related genes	NSCLC HNSCC Melanoma	PD-1/PD-L1 inhibitors (Pembrolizumab, Nivolumab, Atezolizumab, Durvalumab, other)	Objective response rate (complete or partial response) was significantly correlated to tumor microenvironment-related SNPs concerning *CCL2, NOS3, IL1RN, IL12B, CXCR3, and IL6R* genes.	(130)
rs17388568	Metastatic Melanoma	Nivolumab Pembrolizumab	rs17388568 was associated with increased anti-PD-1 response (OR 0.26; 95% CI 0.12–0.53; *p* = 0.0002).	(131)
CD8-, PD-1-and PD-L1-expressing cells	Metastatic Melanoma	Pembrolizumab	Compared to the progression group, the response group was detected with significantly higher numbers of CD8+, PD-1+, and PD-L1+ cells. (CD8, *P* = 0.0001; PD-1, *P* = 0.0002; PD-L1, *P* = 0.006)	(132)
PD-L2	HNSCC	Pembrolizumab	PD-L2–positive patients showed an ORR of 26.5% and PD-L2–negative patients showed an ORR of 16.7%, PD-L2 status was also significantly associated with OS (*P* = 0.030) and PFS (*P* = 0.005)	(133)

Except for the biomarkers mentioned above, the tumor mutation burden/load (TMB) also served as a predictive or prognostic factor for response to anti-PD-1/PD-L1 immunotherapy. TMB is an estimate of somatic mutations by accessing the data from whole exome sequencing (WES) or sequencing a select panel of genes. Foundation Medicine has developed clinical testing platforms to measure TMB using hybrid capture-based next generation sequencing. FDA has approved FoundationOne CDx to be used as a companion diagnostic for therapy selection. Several studies have shown that TMB is associated with a clinical response to anti-PD-1/PD-L1 treatment in melanoma and NSCLC (Table 9). Recently, a novel blood-based TMB (bTMB) assay was developed for cell-free DNA by researchers from Foundation Medicine. A retrospective analysis using bTMB assay showed that bTMB is correlated with significant PFS benefit (*P* = 0.013) and TMB (Spearman rank correlation = 0.64) in patients with NSCLC treated with atezolizumab (139). Neoantigens derived from mutated genes are tumor-specific and show significant correlation with the clinical response to anti-PD-1/PD-L1 treatment. A significantly higher candidate neoantigen burden was detected in patients with CB vs. those with NCB and associated with improved PFS (median 14.5 vs. 3.5 months, log-rank *P* = 0.002) (134). The PFS in patients with a higher non-synonymous burden were higher than those with low non-synonymous burden (median PFS 14.5 vs. 3.7 months, log-rank *P* = 0.01) (134). These data suggested that higher non-synonymous mutation or candidate neoantigen burden in tumors were associated with improved PFS of anti-PD-1-treated NSCLC patients. A recent study has shown that a minority of somatic mutations in tumors could lead to neoantigens and TMB could be used to estimate tumor neoantigen load (140).

**Table 9:** Studies on the predictive effect of TMB on anti-PD-1/PD-L1 immunotherapy.

**Approach for detecting TMB**	**TMB**	**Population**	**Drug**	**Cut-off value**	**Result**	**References**
WES	Non-synonymous mutation burden	NSCLC	Pembrolizumab	High: > 200; Low: < 200.	High non-synonymous burden vs. low non-synonymous burden ORR: 63 vs. 0%; Median PFS: 14.5 vs. 3.7 months *P* = 0.03	(134)
	Non-synonymous mutations in genes on the foundation medicine panel (FM-CGP) and institutional panel (HSLCGP)	Melanoma NSCLC Melanoma	Pembrolizumab	FM-CGP: High: ≥7; Low: <7 HSL-GCP: High: ≥13; Low: <13	CGP-mutational load was significantly associated with progression-free survival (PFS) (FM-CGP *P* = 0.005; HSL-CGP *P* = 0.008). and durable clinical benefit (FM-CGP *P* = 0.03, HSL-CGP *P* = 0.01) in patients treated with PD-1 blockade.	(135)
	Total number of somatic missense mutations	Small cell lung cancer (SCLC)	Nivolumab	Low: 0–<143 mutations; Medium: 143–247 mutations; High: ≥248 mutations.	ORR:High vs. medium vs. low:21.3 vs. 6.8 vs. 4.8% *P* = not reported	(136)
Hybrid capture-based NGS—Foundat-ionOne assay	Hybrid capture NGS panel (315 gene)	Melanoma	Anti PD-1/PD-L1 antibodies (Pembrolizumab, Nivolumab, Atezolizumab)	Low: <3.3 mutations/MB Medium: 0.3–23.1 mutations/MB High: > 23.1 mutations/MB	Mutation load: Initial cohort: Responders vs. non-responders: median 45.6 vs. 3.9 mutations/MB; *P* = 0.003 Validation cohort: Responders vs. non-responders: median 37.1 vs. 12.8 mutations/MB; *P* = 0.002	(137)
	Hybrid-capture-based NGS (182, 236, or 315 genes, depending on the time period)	NSCLC, Melanoma, Other tumors	Anti-PD-1/PD-L1	Low: 1–5 mutations/MB; Medium: 6–19 mutations/MB; High: ≥20 mutations/MB.	High vs. low to medium: RR:58 vs. 20%, *P* = 0.001; PFS:12.8 vs. 3.3 months *P* < 0.0001	(138)

### Discovery of Small Molecule Compounds Inhibiting PD-1/PD-L1 Interactions

The limited success and disadvantage of antibodies prompted researchers to search for more effective strategies for PD-1/PD-L1 targeted therapy and improve the efficacy of cancer immunotherapy. Thus, studies on the discovery of low-molecular-weight compounds inhibiting PD-1/PD-L1 interaction have begun to attract scientist's attention. During the past 5 years, many companies, such as Arising International Inc, Chemocentryx Inc, Institute of Materia Medica, Guangzhou Maxinovel Pharmaceuticals Co, Incyte Corporation, Bristol Myers Squibb (BMS), and Aurigene, have discovered a series of small molecule chemical compounds and peptides.

Meanwhile, these companies have applied for a series of patents related to inhibitors (Table 10). Most of these patents presented not only the structure of PD-1/PD-L1 inhibitors, but also the method of compound synthesis and the use of inhibitors as immunomodulators. In addition, the patents showed verified inhibitory effects of these inhibitors. Some of these inhibitors could only block PD-L1/PD-1 interactions. Other inhibitors, such as the peptides discovered by BMS company, could inhibit interactions of PD-L1 with PD-1 or CD80. All inhibitors discovered by Aurigene, including small molecule chemical compounds and peptides, showed an inhibitory effect on the PD-1 signaling pathway.

**Table 10:** Patents and patent applications of small molecule inhibitors of PD-1 and PD-L1.

**Type**	**Target**		**Patent number**	**Inventor**
Small molecules	PD-1/PD-L1 interaction	Bristol-Myers Squibb Company	WO2015034820A1	Chupak et al. (141)
	Interaction of PD-L1 with PD-1/CD80	Bristol-Myers Squibb Company	WO2015160641A2	Chupak et al. (142)
			WO2018009505A1	Yeung et al. (143)
			WO2017066227A1	Yeung et al. (144)
			WO2018044963A1	Yeung et al. (145)
		Arising International, LLC	WO2018026971A1	Wang et al. (146)
			WO2018045142A1	Webber et al. (147)
		Chemocentryx, Inc.	WO2018005374A1	Lange et al. (148)
	PD-1/PD-L1 interaction	Institute of Materia Medica, Chinese Academy of Medical Sciences.	WO2017202275A1	Feng et al. (149)
			WO2017202273A1	Feng et al. (150)
			WO2017202276A1	Feng et al. (151)
		Guangzhou Maxinovel Pharmaceuticals Co., Ltd	WO2018006795A1	Wang et al. (152)
	PD-1 signaling pathway.	Aurigene Discovery Technologies Limited.	WO2016142852A1	Sasikumar et al. (153)
			WO2016142894A1	Sasikumar et al. (154)
			WO2015033301A1	Sasikumar et al. (155)
			WO2015033299A1	Sasikumar et al. (156)
			WO2016142886A2	Sasikumar et al. (157)
			WO2016142833A1	Sasikumar et al. (158)
			WO2018051255A1	Sasikumar et al. (159)
			WO2018051254A1	Sasikumar et al. (160)
	PD-1/PD-L1 interaction	Incyte Corporation	WO2017205464A1	Lu et al. (161)
			US20170107216A1	Wu et al. (162)
			WO2017070089A1	Wu et al. (163)
			WO2017106634A1	Wu et al. (164)
			US20170174679A1	Lajkiewicz et al. (165)
			US20180057486A1	Wu et al. (166)
			WO2018013789A1	Yu et al. (167)
			US20170362253A1	Xiao et al. (168)
			WO2017192961A1	Li et al. (169)
		Rijksuniversiteit Groningen	WO2017118762A1	Alexander et al. (170)
Peptides	PD-1 signaling pathway.	Aurigene Discovery Technologies Limited	US9096642B2	Sasikumar et al. (171)
			WO2015036927A1	Sasikumar et al. (172)
			WO2015044900A1	Sasikumar et al. (173)
			US9422339B2	Sasikumar et al. (174)
			WO2015033303A1	Sasikumar et al. (175)
			WO2016142835A1	Sasikumar et al. (176)
	Interaction of PD-L1 with PD-1/CD80	Bristol-Myers Squibb Company	US9308236B2	Miller et al. (177)
			US9879046B2	Miller et al. (178)
			WO2016039749A1	Miller et al. (179)
			WO2017176608A1	Miller et al. (180)
			WO2016077518A1	Gillman et al. (181)
			WO2016100608A1	Sun et al. (182)
			US20170252432A1	Allen et al. (183)
			WO2016126646A1	Miller et al. (184)

BMS has published biphenyl derivatives as immunomodulators, and these are the first reported small compounds inhibiting PD-1/PD-L1 interaction. Interestingly, most of the inhibitory compounds showed IC_50_ values of 1 μM or even 0.018 μM as measured by the PD-1/PD-L1 homogenous time-resolved fluorescence (HTRF) binding assay (141). Further modification of the BMS compounds, such as hydrophobic modifications, enhanced the potency of compounds (lowest IC_50_ = 0.48 nM) (143). Moreover, the introduction of symmetric biaryl scaffolds could also improve binding affinities (lowest IC_50_ = 0.04 nM)(144). Arising International LLC published symmetric or semi-symmetric compounds as immunomodulators (IC_50_ values from 0.1 to 25 μM) (146, 147). ChemoCentryx reported 4-phenyl-2,3-dihydro-1H-inden-1-ol derivatives as inhibitors of the PD-1/PD-L1 interaction (147). The Institute of Materia Medica at the Chinese Academy of Medical Sciences has also discovered a series of bromo benzyl ether derivative and phenylate derivative blocking PD-1/PD-L1 interaction (IC_50_: 1 × 10^−4^ nM−1 nM) (149–151). Guangzhou Maxinovel Pharmaceuticals Co., Ltd reported that aromatic acetylene or aromatic ethylene compounds had a significant inhibitory effect on PD-1 and PD-L1 (152). A series of oxadiazole- and thiadiazole- compounds have been developed to inhibit the PD-1/PD-L1 pathway by Aurigene Discovery Technologies Limited (153–160). Incyte Corporation identified a series of heterocyclic compounds as inhibitors for PD-1/PD-L1 protein/protein interaction (IC_50_ values range from the nanomolar to micromolar) (161–169). Meanwhile, Aurigene Discovery Technologies Limited has designed a series of tripeptide peptidomimetics and developed cyclopeptides and macrocyclic-peptides based on peptidomimetics (171–176). Furthermore, BMS developed a series of macrocyclic peptides against the PD-1/PD-L1 pathway (177–184).

However, the discovery of PD-L1/PD-1 inhibitors has only just started. Nearly all inhibitors are still being investigated in preclinical studies. Only CA-170, a PD-L1 inhibitor discovered by Aurigene and Curis, has entered Phase I clinical trial (No: NCT02812875). This has shown acceptable safety of CA-170 (185). The phase II study of CA-170 showed a positive response in two patients with Hodgkin's lymphoma, and the clinical benefit rate is 68.18% (186). Due to its short half-life (6–8 h) vs. other long-lasting antibodies, CA-170 showed less sequalae after being permanently discontinued (186). In addition, preclinical data of the compound CCX4503, published by ChemoCentryx, markedly reduced tumor growth in a human melanoma/peripheral blood mononuclear cell co-implantation model. This preclinical result suggested that the small molecule inhibitors may offer effective anti-tumor therapy (187).

## Discussion and Perspective

Anti-PD-1/PD-L1 antibodies have achieved success in the field of cancer immunotherapy during the past decade and mark a breakthrough in oncology. Eight antibodies blocking PD-1 and PD-L1 interactions have been approved for several indications. Despite the promising results reported in some clinical trials, limited drug efficacy caused by IRAEs has been observed and durable responses have been found in only a limited number of patients. In addition, immune-related adverse events caused by anti-PD-1 drugs have been reported in several clinical trials. Due to the limited successes and disadvantages of anti-PD-1/PD-L1 antibodies, more attention has been given to developing more effective strategies to improve clinical response rates. However, using PD-L1 expression as a biomarker of response is important in identifying patients who could obtain a positive clinical response from PD-1/PD-L1 targeted immunotherapy. The use of a single PD-L1 IHC assay with immunotherapy using a specific anti-PD-1/PD-L1 antibody would be one strategy for improving clinical trial outcomes. However, responses were also seen in patients with negative or low PD-L1 expression. For example, in three trials (CheckMate 017, CheckMate 025, and OAK), favorable long-term outcomes were achieved in PD-L1-negative patients (26, 188, 189). The CheckMate 227 trial among NSCLC patients with a high tumor mutational burden showed that progression-free survival was significantly longer with first line nivolumab plus ipilimumab than with chemotherapy, regardless of PD-L1 status (190). These studies also suggested that a higher mutation or neoantigen load could potentially result in a higher likelihood of response to PD-1 or PD-L1 inhibitors. Apart from TMB, there are several other biomarkers including LDH, MMR-deficiency, gene alteration, and IFN-γ related gene. These are useful biomarkers for the response to anti-PD-1/PD-L1 cancer therapy in solid tumors. Some studies have shown dynamic PD-L1 expression in the tumor cells further limits the feasibility of PD-L1 IHC (191). PD-L1 expression could be regulated through extrinsic and intrinsic signaling pathways such as mitogen-activated protein kinase (MAPK) signaling pathway, Janus kinase/signal transducers and activators of transcription (JAK/STAT) signaling pathway, miRNA-related pathway, as well as IFN-γ and TNF-α (192–194). An understanding of the mechanism of regulation of dynamic PD-L1 expression may be useful for developing novel strategies to improve the efficacy of anti-PD-1/PD-L1 drugs. On the other hand, small molecules are expected to reduce immune-related adverse events and promote higher efficacy. Studies on small molecule PD-1/PD-L1 inhibitors have just begun within the preclinical stage. CA-170 is the first PD-1/PD-L1 inhibitor successfully entering clinical trial, and it is potentially a small molecule PD-1/PD-L1 inhibitor in cancer therapy. Future clinical trial results of CA-170 would be important for developing small molecule inhibitors.

## Author's Note

This review has made a summary about clinical studies and patent application of PD-1/PD-L1 targeted therapies. The paper has also shown the promising result of anti-PD-1/PD-L1 drug in various cancer types and several kinds of strategies improving efficacy of anti-PD-1/PD-L1 drug have been mentioned in the paper, including developing companion PD-L1 test, searching for biomarkers, and discovering small molecule PD-1/PD-L1 inhibitors. The paper has shown the development of anti-PD-1/PD-L1 therapies and provided broad knowledge of PD-1/PD-L1 targeted therapies.

## Author Contributions

LG, RW, and HK contributed conception and design of the review article. LG organized the database collection. LG and RW wrote the first draft of the manuscript. HK wrote and revised sections of the manuscript. All authors contributed to manuscript revision, read and approved the submitted version.

## Conflict of Interest

The authors declare that the research was conducted in the absence of any commercial or financial relationships that could be construed as a potential conflict of interest.
